# Formation of Oligovesicular Vesicles by Micromanipulation

**DOI:** 10.3390/membranes1040265

**Published:** 2011-09-26

**Authors:** Yukihisa Okumura, Tohru Ohmiya, Toshiki Yamazaki

**Affiliations:** Department of Chemistry and Material Engineering, Faculty of Engineering, Shinshu University, 4-17-1 Wakasato, Nagano 380-8553, Japan; E-Mails: tohmiya@mc84.shinshu-u.ac.jp (T.O.); tyamaza@mc84.shinshu-u.ac.jp (T.Y.)

**Keywords:** electroformation, electroswelling, giant vesicles, giant liposomes, oligovesicular vesicles, micromanipulation, microvesiculation

## Abstract

Cell-sized lipid bilayer membrane vesicles (giant vesicles, GVs) or semi-vesicles were formed from egg yolk phosphatidylcholine on a platinum electrode under applied electric voltage by electroformation. Micromanipulation of the semi-vesicle by first pressing its membrane with a glass microneedle and then withdrawing the needle left a GV in the interior of the vesicle. During the process, an aqueous solution of Ficoll that filled the needle was introduced into the newly formed inner vesicle and remained encapsulated. Approximately 50% of attempted micromanipulation resulted in the formation of an inner daughter vesicle, “microvesiculation”. By repeating the microvesiculation process, multiple inner GVs could be formed in a single parent semi-vesicle. A semi-vesicle with inner GVs could be detached from the electrode by scraping with a microneedle, yielding an oligovesicular vesicle (OVV) with desired inner aqueous contents. Microvesiculation of a GV held on the tip of a glass micropipette was also possible, and this also produced an OVV. Breaking the membrane of the parent semi-vesicle by micromanipulation with a glass needle after microvesiculation, released the inner GVs. This protocol may be used for controlled formation of GVs with desired contents.

## 1. Introduction

Giant lipid membrane vesicles (GVs) have been used as a membrane model in various biophysical/biochemical studies and in the construction of a membrane-based microchemical system known as an artificial cell [1,2,3]. As a native biological cell possesses various membranous organelles, sophistication of microchemical systems will demand an advanced membrane model with structural subcomponents. GVs that are encapsulating one or more smaller GVs, oligovesicular vesicles (OVVs) [4], can be a good candidate for such a model.

The authors recently reported the construction of an OVV with heterogeneous inner aqueous compartment [5]. In the study, transformation from a GV to an OVV was induced by low concentration of various substances, such as neutral phosphate buffer or glucose, and this phenomenon was used in one of the key steps in the OVV construction. The protocol has the advantage of producing many OVVs at one time. It needs prolonged incubation (approximately 3 h), and the procedure also demands that OVVs should conform to the conditions required for the GV-to-OVV transformation. Another versatile method of controlled formation of OVVs would greatly benefit the construction of an advanced membrane model.

Micromanipulation of GVs is already known [2,6,7,8,9,10,11,12,13,14,15,16,17], and recent sophistication of the technique allows the construction of complex systems, such as networks of GVs connected with lipid nanotubes [7,8,13,14]. Microinjection into GVs that are obtained by electroformation is also well recognized [2,10,11,12]. On the other hand, the use of micromanipulation for controlled formation of OVVs is less studied. In the present study, we investigated the production of OVVs by micromanipulation of GVs or semi-vesicles.

## 2. Results and Discussion

### 2.1. Electroformation of GVs

GVs were formed using a standard electroformation chamber with two parallel thin platinum wires as electrodes. Phospholipid (1-palmitoyl-2-oleoylphosphatidylcholine (POPC)) was deposited on the electrodes as thin film. After filling the chamber with ultrapure water, sinusoidal AC voltage was applied between the electrodes. The conditions were similar to that used in microinjection into GVs by others [10].

**Figure 1:**
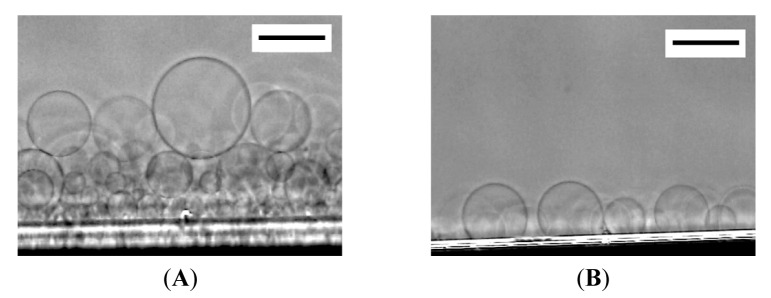
Electroformation of giant vesicles (GVs) from phospholipid deposit on a Pt wire electrode in ultrapure water at 120 min after the application of ac voltage (2.0 Vpp, 10 Hz). Images are GVs formed from relatively thick lipid deposit (**A**), and semi-spherical “mushrooms” from thin deposit (**B**). The electrode appears as the black shadow at the bottom of the frame. Bar = 50 μm.

At 120 min after the application of electric voltage, many spherical GVs of 20–60 μm in diameter were seen on relatively thick lipid deposit (Figure 1A). At the place where the lipid deposit was thin, semispherical “mushrooms” were formed (Figure 1B) [10].

### 2.2. Microinjection into Large Membrane Vesicles

**Figure 2:**
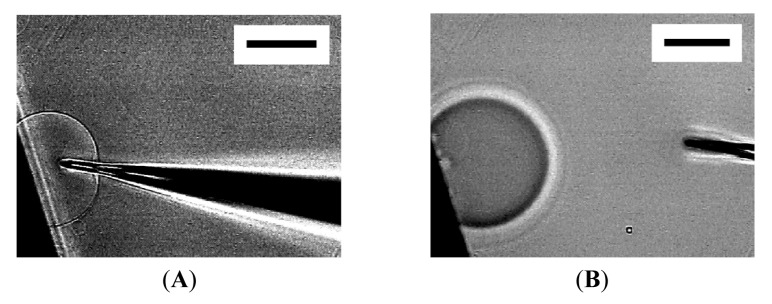
Microinjection into semi-spherical membrane. Images are a semi-sphere with a microneedle inserted (**A**), and after injection of Ficoll solution (**B**). Due to the difference in refractive index, the presence of Ficoll makes the object appear dark. The electrode appears as the black shadow at the lower left of the frame. Bar = 50 μm.

Then, micromanipulation to the membranous objects with a glass microneedle filled with Ficoll solution was attempted. The presence of Ficoll can be detected as change in brightness under the phase contrast observation, and the compound was used as a model substance to monitor encapsulation in vesicles. Figure 2 shows the insertion of a microneedle into a mushroom-like semi-vesicle and microinjection. Successful encapsulation of injected Ficoll was observed as reported by others [10]. It should be noted that the size of the semi-sphere slightly increased upon the microinjection [10]. The extra lipid membrane could have come from the nearby lipid deposit on the electrode.

### 2.3. “Microvesiculation” of Large Membrane Vesicles

**Figure 3:**
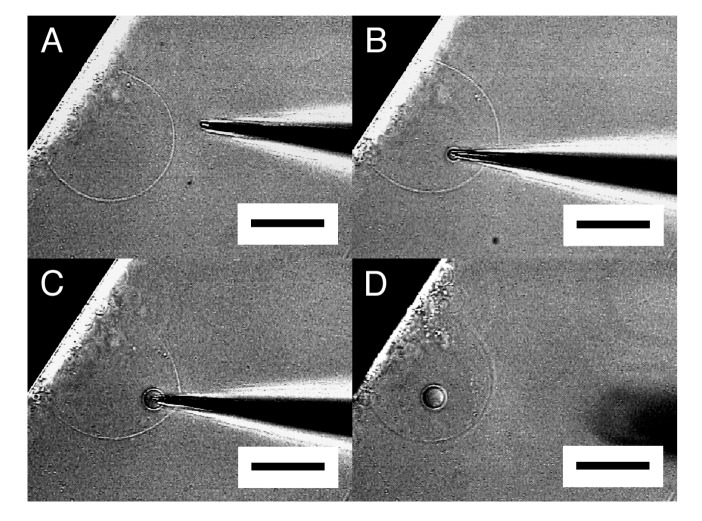
Formation of an inner GV in semi-spherical membrane. Images are the membrane before micromanipulation (**A**), with a microneedle inserted (**B**), with bleeding Ficoll solution from the needle (**C**), and with an inner GV encapsulating the Ficoll left after pulling out the needle (**D**). Bar = 50 μm.

When the tip of a microneedle was pressed against a semi-spherical membrane, the needle sometimes did not break into the membrane. Instead, that part of the membrane caved in with the needle. Then, in a successful attempt, pulling out the needle left a GV in the interior of the semi-sphere (Figure 3). A small amount of Ficoll that was bleeding from the tip of the needle was encapsulated in the newly formed inner GV as can be seen from its dark interior.

**Figure 4:**
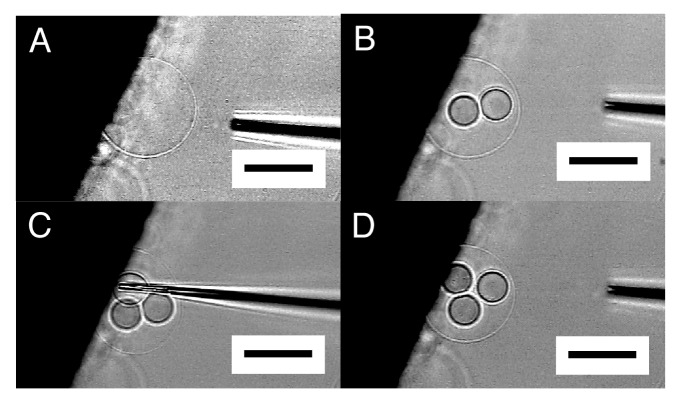
Formation of multiple inner GVs in a single semi-sphere. Images are the semi-sphere before microvesiculation (**A**), two inner GVs already formed by repeating the procedure (**B**), the third GV in formation (**C**), three GVs inside (**D**). Bar = 50 μm.

By repeating this “microvesiculation” process, multiple GVs could be produced in the interior of a single semi-sphere (Figure 4). The structure may be of interest as it has topological resemblance to a biological cell that usually possesses multiple organelles.

It should be noted that the inner GVs seemed to be in contact with one another but did not merge, indicating that those GVs were separated by membrane. In Figure 4, the formation of the three inner GVs hardly changed the size of the outer semi-sphere. In microvesiculation of semi-spherical membrane, a part of the membrane of the inner GVs seems to come from lipid deposit on the electrode, as for microinjection.

**Figure 5:**
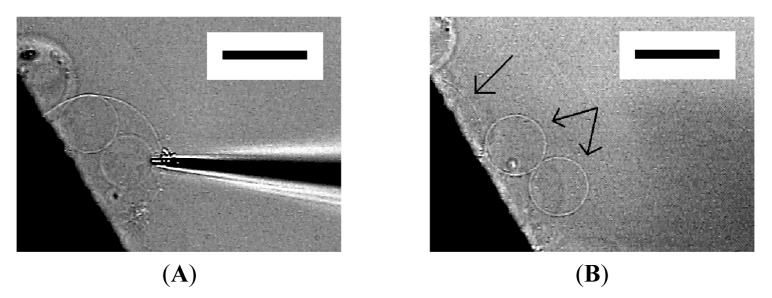
Release of inner GVs upon breaking the outer membrane of a semi-sphere after microvesiculation. Images are a parent semi-sphere with two GVs inside (**A**), and after breaking the outer membrane (**B**). The single arrow indicates the remnant of the broken membrane. Bar = 50 μm.

Breaking the outer membrane by a microneedle after microvesiculation released the inner GVs (Figure 5). This observation indicates that an inner GV is in fact independent of the outer membrane.

The result suggests that the process could also be used as an option for controlled formation of GVs with desired inner contents. With ordinary microinjection, only a small amount of a solution can be introduced into a vesicle because the vesicle has limited capacity and is already almost full. The dilution of the injected solution in the vesicle would be inevitable, and this could complicate the control of the concentration in the vesicle. Meanwhile, preparation of GVs in a solution containing desired substances could encapsulate only a small part of the solution into the GVs, and the efficiency is usually low. In the present case, the whole contents of the formed GV directly came from the solution filled in the microneedle. The concentration should be essentially unchanged, and a minimal amount of the sample would be consumed.

**Figure 6:**
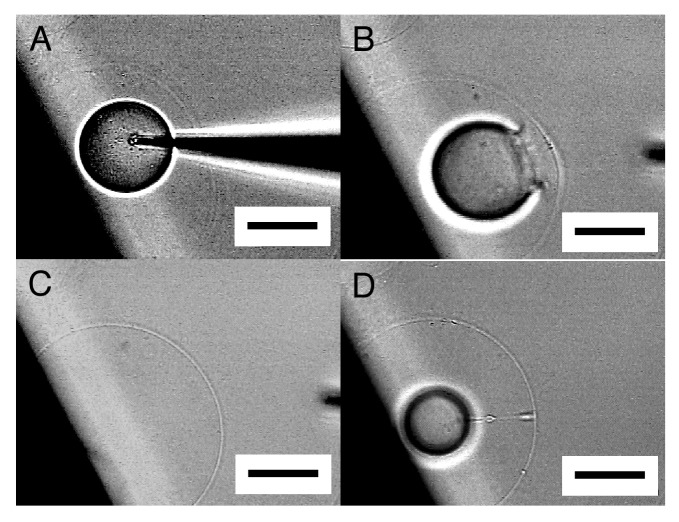
Unsuccessful microvesiculation. Images are a semi-sphere with bleeding Ficoll from inserted microneedle (**A**), immediately (**B**), and 10 s after the needle was pulled out (**C**). Sometimes, a tether connecting the inner and outer membranes could be seen (**D**). Bar = 50 μm.

Occasionally, the membrane did not close, and microvesiculation failed. In Figure 6, an inner vesicle with Ficoll seemed to have formed once, but removing the needle left a cave (Figure 6B). Then, the Ficoll was discharged as the membrane restored its spherical shape (Figure 6C).

Sometimes, a thin tether connecting the inner incomplete sphere and the outer membrane was seen after pulling out a microneedle (Figure 6D). After a while, spontaneous disconnection of the tether occurred, and microvesiculation completed. This observation suggests some similarity in the process between microvesiculation and the vesicle inflation technique developed by Karlsson and coworkers for the construction of networks of interconnected vesicles [7,8]. In the latter technique, a lipid nanotube is pulled out from a mother vesicle by the tip of a microneedle, and then a daughter vesicle is formed by injecting solution through the needle to inflate the membrane at the end of the nanotube. In microvesiculation, a microneedle first pushes the membrane of a mother vesicle into the interior to produce a cave, then bleeding Ficoll solution through the needle inflates the membrane at the closed end of the cave, and removal of the needle leaves a daughter vesicle with a tether.

**Figure 7:**
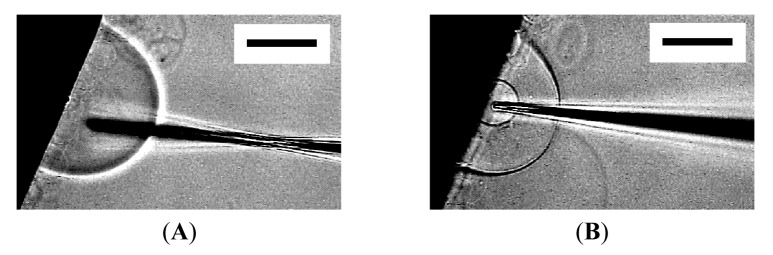
Microinjection and microvesiculation to a single semi-sphere. After microinjection with a microneedle (**A**), the needle was pulled out. Then the same needle was reinserted, and this time, microvesiculation occurred (**B**). Bar = 50 μm.

Under the conditions of the present study, approximately 50% of attempted micromanipulation on semi-spheres resulted in successful microvesiculation. Very subtle difference in the conditions seems to decide between microvesiculation and microinjection. In fact, a single semi-sphere could first be the subject of microinjection and then underwent microvesiculation by using the same needle (Figure 7).

This observation suggests that the factor deciding between microvesiculation and microinjection could not simply be such things as the preparation of membrane or a microneedle. Membranes produced by electroformation under various electrical conditions, along with microneedles of different diameters or cut angles of their tips, were tested (data not shown), but no apparent correlation between those factors and the mode of micromanipulation has been seen. We are presently looking further into this point. Meanwhile, this uncertainty in microvesiculation should usually cause little problem in application of the procedure for construction of a plain oligovesicular system. There are many semi-spheres or GVs available, and after repeating the attempt a few times, one may be able to have a satisfactory result.

### 2.4. Oligovesicular Vesicles by Microvesiculation

**Figure 8:**
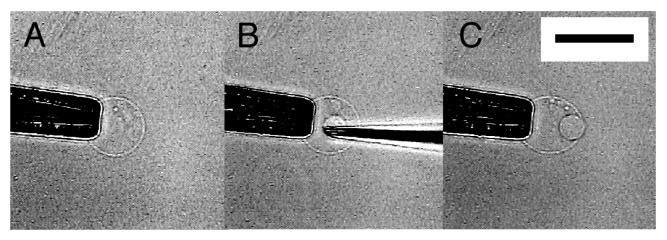
Formation of an OVV by microvesiculation of a GV. Images are a GV held at the tip of a pipette before micromanipulation (**A**), with a microneedle inserted (**B**), and an OVV formed (**C**). Bar = 50 μm.

Unlike semi-spheres that are firmly immobilized, micromanipulation of spherical GVs formed at the top of lipid deposit (Figure 1, left) was difficult because the GVs often escape from the needle tip when they are pressed against it. By detaching a GV from the lipid deposit and holding it on a pipette, microvesiculation to the spherical GV was possible, resulting in an OVV (Figure 8). However, with this procedure, most attempted micromanipulation became microinjection, and approximately only 10% of attempts resulted in successful microvesiculation. Other than possible difference in the characteristics between a semi-sphere on an electrode and a GV on the tip of a glass pipette, lack of the lipid source of an inner GV membrane could be a reason. Similar necessity of a lipid source was also seen in the construction of networks of GVs and lipid tethers by the vesicle inflation technique [7,8].

**Figure 9:**
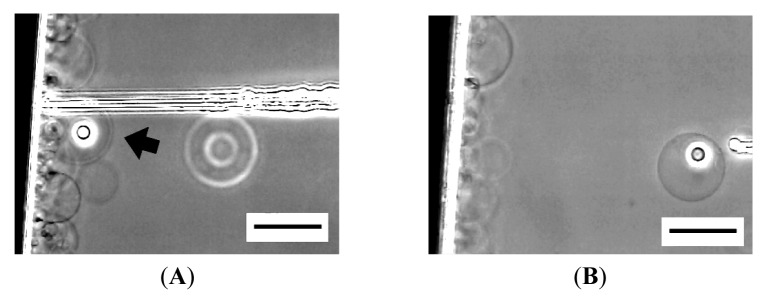
Formation of an OVV from a semi-spherical membrane by physical detachment from the electrode by a microneedle. Images are a semi-sphere before (**A**, arrow) and after (**B**) detachment by using a microneedle. Bar = 50 μm.

A more practical way to obtain an OVV is scraping off a semi-sphere after microvesiculation from an electrode by using a microneedle (Figure 9). Upon the detachment, the semi-sphere closed to an OVV. This approach does not require the extra step of holding a GV. More importantly, the protocol allows multiple operations on a single firmly attached semi-sphere before the final detachment and the formation of an OVV.

One of the essential points for an OVV to be an advanced membrane model is that the interior aqueous phase of its inner GV has desired contents. The OVV obtained in this study by microvesiculation and scraping has designated contents in the inner GV, in the present case, Ficoll as a model substance, and fulfills the requirement.

## 3. Experimental Section

### 3.1. Materials

1-Palmitoyl-2-oleoylphosphatidylcholine (POPC) was purchased from Avanti Polar Lipids (Alabaster, AL, USA). The phospholipid was checked with thin layer chromatography on a silica gel plate (Silicagel 70 Plate-Wako from Wako Pure Chemicals (Osaka, Japan)) developed using chloroform/methanol/water (65:25:4, v/v/v), and only a single spot was seen. Platinum wire (purity 99.98%, diameter 0.50 mm) was obtained from Nilaco (Tokyo, Japan). Methanol and diethyl ether were of the analytical grade and products of Wako Pure Chemicals. Ficoll 400 was a product of Pharmacia Biotech (Uppsala, Sweden).

### 3.2. Electroformation of Vesicles

Two parallel thin platinum wires (diameter 0.5 mm, separation 5 mm) were placed at 0.5 mm from the bottom of a round polystyrene well (inner diameter 40 mm, thickness 5 mm) that had a square glass observation window (15 mm × 15 mm) at the bottom. The conditions of electroformation were similar to that used in microinjection into GVs by others [10]. A solution of POPC dissolved in a mixture of diethyl ether and methanol (9:1 by volume) was carefully deposited on the electrode as a tiny droplet (0.50 μL, 0.5 mg lipid/mL). After spontaneous evaporation of the organic solvent, the lipid deposit was further dried under reduced pressure using a water aspirator for 2 h. The formation chamber was then filled with Milli-Q grade ultrapure water, and sinusoidal ac voltage (2.0 Vpp, 10 Hz) was applied between the electrodes from a function generator (Kenwood TMI FG-272, Yokohama, Japan). Formation of GVs was observed on an inverted optical microscope equipped with phase contrast options and a digital image enhancement system (Olympus IX-50, Tokyo, Japan).

### 3.3. Micromanipulation of Vesicles

A hollow needle was prepared from a borosilicate glass capillary with filament (Narishige GD-1, Tokyo, Japan; the outer and inner diameters were 1.0 mm and 0.6 mm, respectively) using a puller (Narishige PC-10, Tokyo, Japan). The tip of a typical microneedle had the outer diameter of approximately 5 μm and the cut angle of 35 degrees. The needle was attached to a microinjector (Narishige IM-300, Tokyo, Japan) and filled with an aqueous solution of Ficoll 400 (40 mg/mL).

Microvesiculation was attempted 30 times, and the success rate was calculated. Two persons independently tried the procedure, and the rates were similar.

## 4. Conclusions

The present study demonstrates a mode of micromanipulation, “microvesiculation,” on a membranous semi-sphere or a GV is useful for the production of an OVV that has desired contents in the inner GV. Such an OVV may be an advanced membrane model in a study of artificial cells. The approach is straightforward and versatile in application. Although the present study used Ficoll as a model substance to be encapsulated in the inner GV for observation purposes, other various substances could similarly be introduced into the interior of the inner GV through the microneedle.

Furthermore, the study showed that repetitive microvesiculation could result in the formation of multiple inner GVs in a single large membranous vesicle. The structure has topological similarity to a biological cell containing multiple organelles.

Another interesting result is the inner GVs formed by microvesiculation of a semi-vesicle can be released upon breaking the outer envelope membrane. The process could be useful as an option for formation of GVs with desired inner contents. Unlike encapsulation by microinjection into a GV or by preparation of a GV in a solution to be encapsulated, the whole contents of the GVs come directly from the solution filled in the microneedle at the time of microvesiculation. The concentration in the GVs should be well-controlled, and consumption of the solution could be minimal.
